# Detection of Microbial 16S rRNA Gene in the Blood of Patients With Parkinson’s Disease

**DOI:** 10.3389/fnagi.2018.00156

**Published:** 2018-05-24

**Authors:** Yiwei Qian, Xiaodong Yang, Shaoqing Xu, Chunyan Wu, Nan Qin, Sheng-Di Chen, Qin Xiao

**Affiliations:** ^1^Department of Neurology & Collaborative Innovation Center for Brain Science, Ruijin Hospital, Shanghai Jiao Tong University School of Medicine, Shanghai, China; ^2^Department of Bioinformatics, Realbio Genomics Institute, Shanghai, China

**Keywords:** neurodegeneration disease, blood, microbiota, 16S rRNA gene, inflammation

## Abstract

Emerging evidence suggests that the microbiota present in feces plays a role in Parkinson’s disease (PD). However, the alterations of the microbiome in the blood of PD patients remain unknown. To test this hypothesis, we conducted this case-control study to explore the microbiota compositions in the blood of Chinese PD patients. Microbiota communities in the blood of 45 patients and their healthy spouses were investigated using high-throughput Illumina HiSeq sequencing targeting the V3-V4 region of 16S ribosomal RNA (rRNA) gene. The relationships between the microbiota in the blood and PD clinical characteristics were analyzed. No difference was detected in the structure and richness between PD patients and healthy controls. The following genera were enriched in the blood of PD patients: *Isoptericola*, *Cloacibacterium*, *Enhydrobacter* and *Microbacterium*; whereas genus *Limnobacter* was enriched in the healthy controls after adjusting for age, gender, body mass index (BMI) and constipation. Additionally, the findings regarding these genera were validated in another independent group of 58 PD patients and 57 healthy controls using real-time PCR targeting genus-specific 16S rRNA genes. Furthermore, not only the genera *Cloacibacterium* and* Isoptericola* (which were identified as enriched in PD patients) but also the genera *Paludibacter* and* Saccharofermentans* were positively associated with disease duration. Some specific genera in the blood were related to mood disorders. We believe this is the first report to provide direct evidence to support the hypothesis that the identified microbiota in the blood are associated with PD. Additionally, some microbiota in the blood are closely associated with the clinical characteristics of PD. Elucidating these differences in blood microbiomes will provide a foundation to improve our understanding of the role of microbiota in the pathogenesis of PD.

## Introduction

Parkinson’s disease (PD) is known to increase with age and currently affects 1% of the population aged >60 years. It is a progressive neurodegenerative disorder characterized by motor and non-motor symptoms (Tysnes and Storstein, 2017). The cause of PD remains unknown. Neuroinflammation is recognized as a key factor in the initiation and progression of PD pathology (Wang et al., 2015). Particularly, systemic and/or neuroinflammation in the brain might result from excessive stimulation of the innate immune system induced by gut microbiota dysbiosis and increased intestinal permeability (Galland, 2014). The microbiota present in feces, including both commensal and pathogenic organisms, may affect brain activity through the gut-microbiota–brain axis under both physiological and pathological conditions (Grenham et al., 2011; Wang and Kasper, 2014). Recently, several studies based on 16S ribosomal RNA (rRNA) gene sequencing have shown consistent alterations in the composition of the gut microbiota, both in early and advanced PD patients (Keshavarzian et al., 2015; Scheperjans et al., 2015; Hopfner et al., 2017; Petrov et al., 2017). The gut microbiota could induce inflammation in both gut and systemic circulation due to lipopolysaccharides (LPS) from pathogenic bacteria and synthesis of pro-inflammatory cytokines (Mulak and Bonaz, 2015) such as interleukin (IL)-1β, IL-6 and tumor necrosis factor (TNF)-α that were elevated in the blood of PD patients (Dufek et al., 2009; Reale et al., 2009).

The microbiota plays a fundamental role in immunity and inflammation of the host immune system, particularly in the systemic circulation (Hakansson and Molin, 2011). Blood in healthy organisms is considered a *sterile* environment owing to lack of proliferating microbes (Potgieter et al., 2015). However, the notion of presence of truly sterile blood in healthy humans has been challenged. Nikkari et al. (2001) found that even “healthy” blood specimens can contain bacterial 16S rDNA. Sequence-based studies have recently uncovered authentic blood microbiomes in some non-communicable diseases, e.g., type II diabetes (Amar et al., 2011b), liver fibrosis (Lelouvier et al., 2016) and cardiovascular disease (Amar et al., 2013). Direct microscopic measurement showed that microbes were in close proximity to the red blood cells (RBCs) of patients with PD (Potgieter et al., 2015). This raises the question of existence of microbiota in the blood and its impact on the role associated with PD. Microbiota is a collective terminology for all microorganisms, generally including not just bacteria, but also microbes such as yeasts, filamentous fungi, archaea and maybe even viruses (Sekirov et al., 2010). The microbial 16S rRNA gene is the most established genetic marker, is widely used for bacterial identification and classification (Janda and Abbott, 2007). However, to our best knowledge, no study has yet investigated the microbiota, mainly bacteria, in the blood of patients with PD using 16S rRNA gene sequencing.

On the basis of that, we hypothesized possible alterations in the microbiota in the blood of PD patients. The association between microbiota and diseases involving interactions between genetics and the environment are understandably quite complex. Couples share more of their gut microbiota than individuals from different households (Song et al., 2013), and thus, the spouses of PD patients could serve as controls to minimize variation caused by diets. Here, we analyzed the microbiota communities detected in the blood of PD patients with those of their healthy spouses using 16S rRNA gene sequencing. Furthermore, we also analyzed the relationships between the microbiota present in the blood of PD patients and the clinical characteristics of PD.

## Materials and Methods

### Ethics Statement

This study protocol was approved by the Research Ethics Committee, Ruijin Hospital, Shanghai Jiaotong University School of Medicine, Shanghai, China. All participants were informed of the purpose of this study and gave written informed consent in accordance with the Declaration of Helsinki.

### Study Subjects

All PD patients involved in this study were diagnosed with PD according to the UK Brain Bank criteria (Daniel and Lees, 1993). Those diagnosed with diabetes, heart failure, liver cirrhosis, malignancy, hematological or autoimmune diseases, and infectious disease were excluded. The healthy controls exhibited no disease symptoms. Individuals currently taking antibiotics within the 3 months prior to sample collection were also excluded. A total of 45 patients (23 [51.1%] female; mean age: 68.1 ± 8.0 years) and their healthy spouses (22 [48.9%] female, mean age: 67.9 ± 8.0 years), who were living in the same household for at least 20 years were enrolled from the Movement Disorders Clinic at the Department of Neurology of Shanghai Ruijin Hospital.

To validate the significantly different genera using real-time PCR, an independent group of 58 PD patients (32 [55.2%] female; mean age: 67.5 ± 7.6 years) and 57 healthy controls (31 [54.4%] female, mean age: 67.0 ± 7.9 years) was also enrolled.

### Clinical Data Collection

All clinical data were collected through face-to-face interviews with movement disorder specialists. All measurements and questionnaires were voluntary. The examining physician measured the weight and height of all subjects, and then calculated the body mass index (BMI). The Unified Parkinson’s Disease Rating Scale (UPDRS) and the Hoehn and Yahr stage (H&Y stage) of patients were examined during the “on” state. Forty-five PD patients for 16S sequencing had an average H&Y stage of 2.2 ± 0.7; age of onset, 62.6 ± 8.1 years; disease duration, 5.7 ± 4.1 years; and UPDRS total scores, 38.4 ± 18.8. Additionally, all PD patients were using antiparkinsonian medications, and no one underwent surgery. Levodopa equivalent doses (LED) were calculated using a classical method according to a previous study (Tomlinson et al., 2010). The average LED of 45 patients was 427.4 ± 259.9 mg/day, and 16 patients (35.6%) were diagnosed with motor complications (diagnosed according to the UPDRS part IVA and IVB; Chapuis et al., 2005). The PD-related non-motor symptoms (NMSs) of patients were evaluated using the Non-Motor Symptoms Questionnaire for PD (NMS-Quest), Hamilton Anxiety Scale (HAMA), Hamilton Depression Scale (HAMD), Mini Mental State Examination (MMSE) and Montreal Cognitive Assessment (MoCA). Constipation was assessed using the Rome III Criteria (Longstreth et al., 2006). Forty-five PD patients had average NMS scores of 6.7 ± 4.0, HAMD scores of 5.5 ± 5.6, HAMA scores of 7.5 ± 6.1, MMSE scores of 29.0 ± 2.0 and MoCA scores of 24.3 ± 4.1.

### Sample Collection and DNA Extraction

Many reagents required in the real-time PCR and sequencing pipeline contain nonnegligible amounts of bacterial DNA, which can be misinterpreted as present in the samples (Salter et al., 2014). The DNA extraction were performed carefully to minimize any risk of contamination between samples or by the researchers. All the DNA extraction were performed by the same person within 3 days. DNA was extracted carefully from peripheral blood leukocytes using a classical phenol/chloroform extraction method (Amar et al., 2011b). After ethanol precipitation, DNA was resuspended in ddH_2_O and stored at −80°C prior to use. All the extractions of DNA were prepared under a Class II biologic safety cabinet. The concentration of genomic DNA in each blood sample was quantified using a NanoDrop 2000 spectrophotometer (Thermo Scientific, MA, USA).

### 16S rRNA Gene Quantification by Real-Time PCR

The 16S rRNA gene copies of each sample was evaluated by real-time PCR using universal forward and reverse primers, EUBF: 5′-TCCTACGGGAGGCAGCAGT-3′ and EUBR: 5′-GGACTACCAGGGTATCTAATCCTGTT-3′. The PCR reactions were performed in a total volume of 10 μl (in a 384-well format) using SYBR green mixture (Takara, Japan) comprising 10 nM (each) forward and reverse primers and 1 μl of sample DNA. The reaction conditions for amplification were 95°C for 10 s and 40 cycles at 95°C for 5 s and 60°C for 30 min, which was followed by the melting curve step according to the manufacturer’s instructions. The standard curve for 16S rRNA gene number quantification was performed by generating a series of 10-fold dilutions from 10^2^ to 10^10^ of 16S rRNA gene copies per reaction using the DNA of *Escherichia coli* BL21 strain. Amplifications of samples and standard dilutions were performed in triplicate on the ABI ViiA7 instruments detection system (Applied Biosystems by Life Technologies, Austin, TX, USA). Compared with microbiota DNA detected in the blood using real-time PCR, the level of no template controls was very low which demonstrated that the overall background signals (from reagents or potential contamination by the experimenter) could be ignored (Supplementary Figure S1).

### 16S rRNA Gene Amplicon and Sequencing

Universal primers (341F and 806R) linked with indices and sequencing adaptors were used to amplify the V3-V4 regions of the 16S rDNA. PCR amplification was performed in 20-μL reactions containing 10× polymerase mix (Life Technologies, Carlsbad, CA, USA), 10 μM of the forward and reverse primers, and 25 ng of template DNA. The amplicon sequencing libraries were sequenced on an Illumina HiSeq platform to obtain 250-bp paired-end reads. The total samples resulted in 5,285,321 clean reads with an average of 58,725.8 ± 6134.8 clean tags per sample. A total of 16,549 tags were calculated from each sample based on the sequencing saturation and integrity.

### Genus-Specific Quantification by Real-Time PCR

The comparison and analysis of the 16S region sequences with those of related genera reference strains were carried out using the multiple alignment program DNAman software was used (version 8.0) to determine the regions conserved only among genera *Limnobacter, Myroides*, *Isoptericola*, *Microbacterium*, *Cloacibacterium* and *Enhydrobacter* (detail reference strains of each genus for assembly are listed in Supplementary Table S3), from which the genus-specific primers were derived. The specific primer target sites for the quantitative analysis were performed using Primer 5. Universal 16S rRNA gene was used as the internal control and an abundance of genus were expressed as relative levels to 16S rRNA. The primers were designed by Realbio Genomics Institute. The PCR reaction and condition were the same with 16S rRNA gene quantification. The genus-specific primer sequences used in this study are listed in Table 1.

**Table 1:** Genus-specific 16S rRNA gene target primers of real-time PCR.

Genus	Primer (5′–3′)	Amplicon size
*Cloacibacterium*	F: GGCGCAAGCTTCAGAGA	117 bp
	R: AACCACATAATCCACCGCTT	
*Enhydrobacter*	F: GGTTCGGCCGGAGTCAA	104 bp
	R: GGCAACTGGAGACGAGGGT	
*Isoptericola*	F: CTCATTCCACGAGTTCCGA	115 bp
	R: ATGCTCCGCCGCTTGT	
*Limnobacter*	F: TGCTCGAAAGAGAACCTGC	128 bp
	R: CTCATTAGAGTGCCCTTTCGTA	
*Myroides*	F: GGTTTTCGGACTGAGTGG	130 bp
	R: ATCATCGAATTAAACCACATGC	
*Microbacterium*	F: GAAGGCATCTTCAGCGG	143 bp
	R: CGTGTCTCAGTCCCAGTGTG	
16S	F: GCTCGTGTCGTGAGATGTT	159 bp
	R: TGTAGCCCAGGTCATAAGG	

### Sequence Analysis

The raw 16S rDNA data were processed to form operational taxonomic units (OTUs) at 97% identity using UPARSE (Edgar, 2013). Taxonomy was assigned using the Ribosomal Database Project (RDP) as the reference database. The α-diversity and β-diversity indices were calculated based on the rarefied OTU counts using the Qiime program. α-diversity represents an analysis of diversity in a single sample reflected by parameters including good coverage, Chao 1, PD whole tree, Shannon index and Simpson index using Qiime (Caporaso et al., 2010). Wilcoxon rank sum test was used to compare each α-diversity index. β-diversity is used as a measure of the microbiota structure between groups. Both the weighted and unweighted Unifrac distance matrices were plotted in the principal coordinate analysis (PCoA), and analyses of similarities (ANOSIMs) were performed using the R package “ade4.” For taxa with a prevalence ≥10%, differential abundance analysis was performed using the Wilcoxon rank-sum test at the phylum, class, order, family, and genus levels. For multiple comparisons of bacterial counts, the false discovery rate (FDR) was calculated using the Benjamini and Hochberg method. Microorganism features used to distinguish the blood microbiotas specific to PD were identified using the linear discriminant analysis (LDA) effect size (LEfSe) method^1^ with an alpha cutoff of 0.05 and an effect size cutoff of 2.0. Phylogenetic Investigation of Communities by Reconstruction of Unobserved States (PICRUSt) was used to predict the abundances of functional categories in the Kyoto Encyclopedia of Genes and Genomes (KEGG) orthologs (KO). The graph of KEGG pathways in level 2 (41 pathways) and level 3 (328 pathways)^2^ was performed with STAMP, and *P* values were calculated with White’s non-parametric *t*-test.

### Statistical Analysis

Both SPSS (ver. 21.0, SPSS Inc., Chicago, IL, USA) and R software (ver. 3.1.0, the R Project for Statistical Computing) were used for statistical analysis. In the descriptive analyses, the mean ± standard deviation (SD) was used for normally distributed continuous variables and the median ± interquartile range (IQR) for continuous variables with skewed distributions. The comparisons of the relative abundance of the genera-detection using real-time PCR of the PD patients and controls was performed using the Wilcoxon rank-sum test. The associations between genera (sequence counts) with a prevalence ≥10% and clinical parameters of PD and healthy groups were evaluated using a generalized linear model (GLM), employing negative binomials depending on the distribution of the target variable using the R package “glmmADMB.” Random forest (RF) models were used to predict disease status based on the identified different taxa at genus level from Wilcoxon rank-sum test using the default parameters of the R implementation of the Boruta algorithm with the package “randomForest” (Kursa and Rudnicki, 2010; Chen et al., 2016). Correlations between genera (sequence counts) and clinical parameters in 45 PD patients (prevalence ≥10%) were calculated using Spearman’s rank-correlation analysis with the R package “cor.test.” The significant genera associated with the variables in Spearman’s correlation analysis were subjected to GLM analysis adjusted for different confounders. The potential confounders were listed as follows: age, gender, BMI, disease duration, H&Y stage, LED, UPDRS total scores, NMS scores, HAMD scores, HAMA scores, MMSE scores and motor complications. The variables were tested for collinearity using the variance inflation factor (VIF) in the “vif” function in the R package “car.” Of the 12 variables, no evidence for collinearity with each variable was detected (VIF < 5). A 10-fold cross-validated Least Absolute Shrinkage and Selection Operator (LASSO) regression was used to select potential confounders for each genus using R package “lars” (Baradaran et al., 2013; Chaturvedi et al., 2017). GLM was used to estimate the significance of individual variables with controlling the selected confounders. *P* < 0.05 was considered statistically significant.

## Results

### Characteristics of the Studied Groups

There was no difference in age, gender, or BMI between the two groups (Table 2). As expected, a higher proportion of the PD group reported constipation than the healthy group (53.3% vs. 6.7%, respectively, *P* < 0.001, Pearson’s Chi-square test). There was no significant difference in the 16S rRNA gene copies between PD and healthy groups by real-time PCR (1.22E+04 ± 1.21E+05 copies/ng of PD vs. 7.78E+03 ± 6.86E+04 copies/ng of controls, *P* = 0.316, Wilcoxon rank-sum test analysis).

**Table 2:** Characteristics of the study subjects.

Characteristics	PD group	Healthy group	*P* value
	(*n* = 45)	(*n* = 45)	
Age (years)^a^	68.1 (8.0)	67.9 (8.0)	0.875
Female (n, %)	23 (51.1%)	22 (48.9%)	0.903
BMI (kg/m^2^)^a^	22.8 (2.3)	23.4 (2.1)	0.164
Constipation (n, %)	24 (53.3%)	3 (6.7%)	<0.001
16S gene (copies/ng)^b^	1.22E+04 (1.21E+05)	7.78E+03 (6.86E+04)	0.316

*Data are shown as mean (SD)^a^ or median (IQR)^b^. Comparisons between the two groups were performed with Student’s t-test (age and BMI) and Pearson’s Chi-square test (female and constipation), respectively. The 16S gene copies were normalized to 1 ng of each DNA sample, and the comparison between the two groups was performed with Wilcoxon rank-sum test analysis. PD, Parkinson’s disease; BMI, Body Mass Index*.

### Microbiota Present in the Blood of PD and Healthy Groups

The taxonomic diversity and profiles of the microbiota DNA were analyzed by performing high-throughout 16S rRNA gene sequencing. No differences were detected in either the α-diversity or the β-diversity indices of the blood microbiota between the two groups (Supplementary Figures S2A,B), suggesting that no difference was detected in the structure and richness between PD patients and healthy controls.

**Figure 1:**
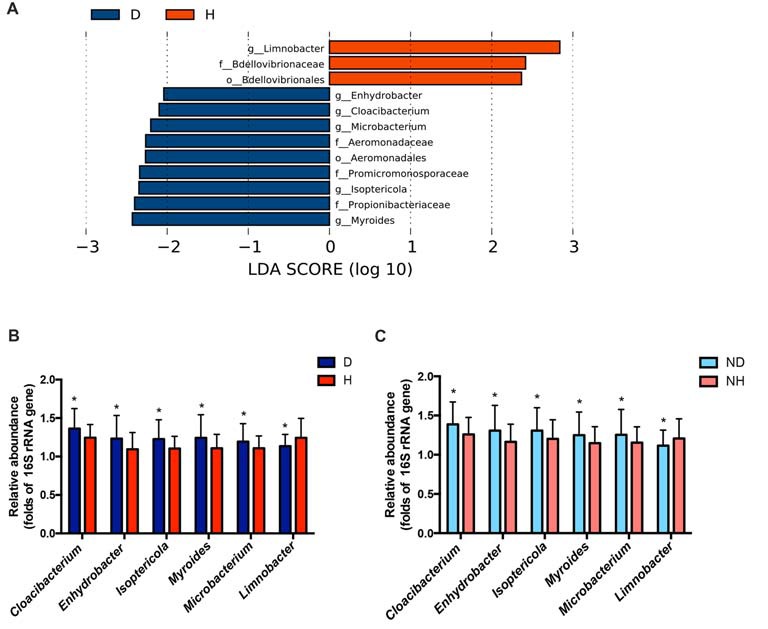
Taxonomic differences in the microbial 16S ribosomal RNA (rRNA) gene in the blood of Parkinson’s disease (PD) and healthy groups. **(A)** Linear discriminant analysis (LDA) effect size (LEfSe) analysis revealed significant bacterial differences in blood microbiota between the PD (negative score) and healthy groups (positive score). The LDA scores (log 10) > 2 and *P* < 0.05 are listed. **(B)** The relative abundance of the genera *Cloacibacterium*, *Enhydrobacter*, *Isoptericola*, *Myroides*, *Microbacterium* and *Limnobacter* between the PD and healthy groups were detected using real-time PCR. The mean ± standard deviation (SD) values were shown for each genus. **(C)** The relative abundance of the genera *Cloacibacterium*, *Enhydrobacter*, *Isoptericola*, *Myroides*, *Microbacterium* and *Limnobacter* were validated in another independent group of 58 PD patients and 57 healthy controls using real-time PCR. The mean ± SD values were shown for each genus. D, PD group (blue); H, healthy group (red); ND, new PD patients (light blue); NH, new healthy controls (light red); p, phylum; c, class; o, order; f. family; g, genus; RF, Random Forest; ROC, receiver operating characteristic; AUC, area under the ROC curve; CI, confidence interval. *P < 0.05 vs. healthy group/controls.

A total of 29 taxa with differential abundances between the PD and healthy groups were identified in the blood microbiota (*P* < 0.05, Wilcoxon rank-sum test analysis, Supplementary Table S1), and no significantly different genus was detected between the two groups at an FDR of 5%. Within LEfSe analysis, greater proportions of the genus *Limnobacter* were detected in the healthy group than in the PD group. Higher proportions of the genera *Myroides, Isoptericola, Microbacterium*, *Cloacibacterium* and *Enhydrobacter* were observed in the PD patients (LDA Score (log10) > 2, Figure 1A). The differences in the blood microbiota between the PD and healthy groups were associated with the genera *Isoptericola*, *Cloacibacterium*, *Enhydrobacter*, *Microbacterium* and *Limnobacter* according to the GLM model (*P* < 0.05, Table 3).

**Table 3:** GLMs for the different genera controlling for other confounders between the PD and healthy groups.

Study group	Genus	*b* value	95% CI	*P* value
	*Isoptericola*	2.309	1.174–3.444	<0.0001
	*Cloacibacterium*	1.360	0.615–2.110	0.0003
PD vs. Healthy	*Enhydrobacter*	1.363	0.603–2.121	0.0004
	*Microbacterium*	0.590	0.075–1.110	0.025
	*Limnobacter*	−0.712	−1.347–0.077	0.028

*Result of the GLMs for significant genera (sequence counts) based on the group factors (PD and healthy group) and possible confounding factors (age, gender, BMI and constipation) of 90 individuals. The b value positive (negative) mean the genus was associated with the PD (healthy) group. GLM, general linear model; CI, confidence interval; BMI, body mass index*.

Particularly, we designed the genus-specific primers and performed real-time PCR to examine the changes of these genera in both PD patients and healthy controls. The higher relative abundances of the genera *Cloacibacterium*, *Enhydrobacter*, *Isoptericola*, *Myroides*, *Microbacterium*, and lower relative abundances of the genera *Limnobacter* were detected in PD patients using real-time PCR (*P* = 0.0156, 0.0265, 0.0134, 0.0200, 0.0391 and 0.0431, Wilcoxon rank-sum test analysis, Figure 1B). Furthermore, we tested these genera in the blood of another independent group of 58 PD patients and 57 healthy controls to validate the results, and the same alterations of these genera were detected (*P* = 0.0310, 0.0395, 0.0368, 0.0273, 0.0206 and 0.0262, respectively, Wilcoxon rank-sum test analysis, Figure 1C).

**Figure 2:**
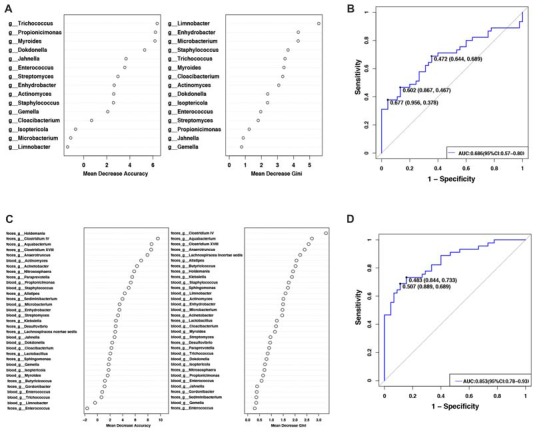
Predictive model based on the genus-level abundance profile in the blood or combination of feces and blood, using RF.** (A)** The predictive model based on genus-level abundance taxa in the blood using an RF model. The relative importance of each genus in the predictive model was performed using the mean decreasing accuracy and the Gini coefficient for microbiota. **(B)** ROC curve generated by the RF using 15 genera from the microbiota. The plots shown in the ROC represent the corresponding optimal threshold. **(C)** The relative importance of each genus in the predictive model was assessed using mean decreasing accuracy and Gini coefficient in the fecal-blood combined microbiota. **(D)** ROC curve generated by the RF model using 33 genera fecal-blood combined microbiota. The plots shown in ROC indicate the corresponding optimal threshold (sensitivity, specificity) with the AUC. ROC, Receiver operating characteristic; AUC, area under the ROC curve; CI, confidence interval.

To evaluate PD disease status based on an ensemble of decision trees, we used RF to build a predictive model. A total of 15 genera were considered to be predictive of disease (Figure 2A). A mean classification error of 0.38 was achieved, and the area under the receiver operating characteristic curve (AUC) was 0.686 (95% confidence interval (CI): 0.57–0.80, sensitivity 37.8% and specificity 95.6% with a cut-off rate of 67.7%, Figure 2B). Genus *Limnobacter* was the most important and accurate taxon among the identified taxa between the two groups (Figure 2A). Recently, the fecal microbiota community of these same 90 individuals were also detected (Qian et al., 2018). Combining the taxa from the feces and blood, 33 genera were identified to be predictive of PD (Figure 2C), achieving a mean classification error of 0.23 and an AUC of 0.853 (95% CI: 0.78–0.93, sensitivity 68.9% and specificity 88.9% with a cut-off rate of 50.7%, Figure 2D).

**Figure 3:**
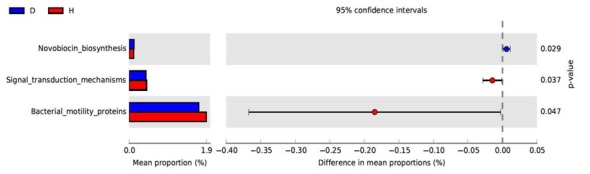
Functional predictions of microbiota present in the blood of PD patients and healthy controls. Significant KEGG pathways of Level 3 for the microbiome of the PD and healthy groups was identified by STAMP software. In STAMP, differences in abundances between the PD and healthy groups were compared using White’s non-parametric *t*-test. CIs were estimated using a percentile bootstrapping method (10,000 replications). D, PD group (blue); H, healthy group (red); KEGG, Kyoto Encyclopedia of Genes and Genomes; KO, KEGG orthologs; PICRUSt, Phylogenetic Investigation of Communities by Reconstruction of Unobserved States.

PICRUSt based on closed-reference OTU was used to predict the abundances of functional categories KO. In all, 98 significantly different KOs were detected in the microbiome of the two groups (*P* < 0.05, Wilcoxon rank-sum test analysis, data not shown). However, no significantly different KOs was identified at an FDR of 5%. None of the significant pathways were identified in the level 2 of KEGG. In the level 3 KEGG pathways, the microbial gene function related to novobiocin biosynthesis was increased in the blood of PD patients, whereas signal transduction mechanisms and bacterial motility proteins were lower in PD patients than in healthy controls (*P* < 0.05, Figure 3).

### Clinical Association Between the Microbiota and PD Characteristics

**Figure 4:**
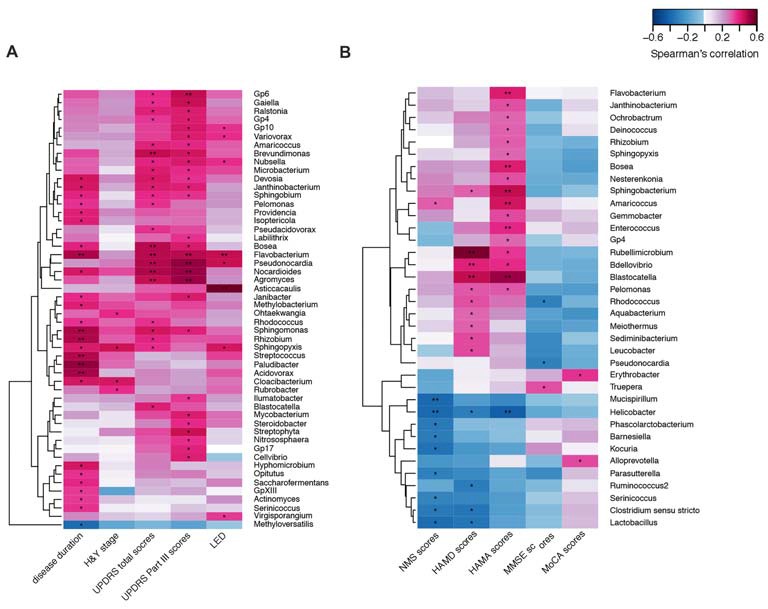
Correlations between the genera and PD clinical characteristics using heatmaps. The correlations between the microbiota present in the blood based on the abundance (sequence counts) of the genus (prevalence ≥10% in PD patients) and PD clinical characteristics, particularly disease duration, severity, medication (LED)** (A)**, and non-motor symptoms **(B)** were performed with heatmaps using Spearman test. PD severity includes H&Y stage and UPDRS total and part III scores. Non-motor symptoms of PD are represented by NMS scores, depression (HAMD scores), anxiety (HAMA scores), and cognitive impairment (MMSE and MoCA scores). The intensity of the color represents the *r* value (correlation; negative score, blue; positive score, red). H&Y stage, Hoehn and Yahr stage; UPDRS, Unified Parkinson’s Disease Rating Scale; LED, levodopa equivalent doses. NMS, non-motor symptoms; HAMD, Hamilton Depression Scale; HAMA, Hamilton Anxiety Scale; MMSE, Mini Mental State Examination; MoCA, Montreal Cognitive Assessment. UPDRS scores were obtained during the on-phase at the outpatient clinic. Spearman test, *P < 0.05, **P < 0.01.

We confirmed a correlation between the microbiota (genus level, at a prevalence ≥10%) present in the blood and PD clinical parameters, particularly disease duration, PD severity (H&Y stage, UPDRS total and part III scores), and medication (LED). Most of the identified genera in the blood exhibited a positive correlation (Figure 4A), e.g., *Flavobacterium* with disease duration; total UPDRS and part III scores and LED; and *Pseudonocardia*, *Nocardioides* and *Agormyces* with UPDRS total and part III scores (*P* < 0.01). To find out significant taxa associated with the clinical variables, the GLM method was performed to adjust the potential confounders selected by the LASSO regression. Besides genera *Cloacibacterium* and* Isoptericola*, which were identified as enriched among PD patients, *Paludibacter and Saccharofermentans* were positively associated with disease duration. Genera *Janibacter*, *Nocardioides* and* Pseudonocardia* were positively associated with UPDRS total scores. Genera *Asticcacaulis* and* Sphingopyxis* were positively associated with LED (Table 4). Although 12 significantly different taxa in blood were identified in PD patients with motor complications (*P* < 0.05), none of these taxa were significantly different at an FDR of 0.05 (Supplementary Table S2). After adjusting the possible confounders, genus *Dokdonella* was enriched in patients with motor complications (Table 4).

**Table 4:** Summary of GLM results for correlations between the taxon with the PD characteristics in patients.

PD characteristics	Study group	Genus	*b* value (95%CI)	*P* value
	disease duration	*Cloacibacterium*	0.151 (1.051–1.287)	0.003
		*Isoptericola*	0.377 (1.272–1.671)	<0.0001
		*Paludibacter*	0.318 (1.223–1.543)	<0.0001
		*Saccharofermentans*	0.552 (1.419–2.127)	<0.0001
Disease duration and medication of PD	UPDRS total scores	*Janibacter*	0.051 (1.028–1.076)	<0.0001
		*Nocardioides*	0.033 (1.008–1.060)	0.010
		*Pseudonocardia*	0.023 (1.001–1.046)	0.040
	LED	*Asticcacaulis*	0.004 (1.003–1.007)	<0.0001
		*Sphingopyxis*	0.002 (1.000–1.004)	0.011
Motor complication	motor complications	*Dokdonella*	−1.576 (0.048–0.893)	0.035
	NMS scores	*Kocuria*	−0.164 (0.750–0.959)	0.009
		*Parasutterella*	−0.477 (0.485–0.842)	0.001
		*Phascolarctobacterium*	−0.618 (0.7200–0.992)	0.040
Non-motor symptoms of PD	HAMA scores	*Amaricoccus*	0.110 (1.036–1.203)	0.004
		*Bosea*	0.060 (1.015–1.112)	0.0098
		*Janthinobacterium*	0.104 (1.041–1.182)	0.001
		*Nesterenkonia*	0.067 (1.012–1.129)	0.017
		*Sphingobacterium*	0.161 (1.092–1.262)	<0.0001
	HAMD scores	*Aquabacterium*	0.082 (1.024–1.153)	0.006
		*Bdellovibrio*	0.220 (1.061–1.463)	0.007
		*Leucobacter*	0.097 (1.037–1.170)	0.002

*Instruction: these results of GLM were performed with significant correlated genus (from Spearman’s test) as the dependent variable and the study group as the independent variables. For briefly, we listed the significant independent variables (at the second column of the left in the table) and classified them with different clinical meanings (the first column of the left in the table). A 10-fold cross-validated LASSO regression method was used to select variables for each genus. The potential confounders were listed as follows: age, gender, BMI, disease duration, H&Y stage, LED, UPDRS total scores, NMS scores, HAMD scores, HAMA scores, MMSE scores, and motor complications. Result of the GLM for significant (Spearman, *P* < 0.05) genus (sequence counts) based on the group factors and possible confounders. LASSO, Least Absolute Shrinkage and Selection Operator; H&Y stage, Hoehn and Yahr stage; UPDRS, Unified Parkinson Disease Rating Scale; LED, Levodopa Equivalent Doses; NMS, Non-Motor Symptoms; HAMD, Hamilton Depression Scale; HAMA, Hamilton Anxiety Scale; MMSE, Mini Mental State Examination; MoCA, Montreal Cognitive Assessment; GLM, general linear model; CI, confidence interval*.

We also discovered the correlations between the taxa and NMSs, e.g., genera *Helicobacter* and* Mucispirillum*, negatively correlated with NMS scores (*P* < 0.01 Figure 4B). Most of the correlated genera in the blood were positively associated with mood disorders, e.g., genera *Rubellimicrobium, Bdellovibrio* and *Blastocatella* with HAMD scores and genera *Flavobacterium*, *Bosea*, *Sphingobacterium*, *Amaricoccus*, *Enterococcus*, *Blastocatella* and *Helicobacter* with HAMA scores (*P* < 0.01, Figure 4B). After adjusting for confounding factors, genera *Kocuria*, *Parasutterella* and *Phascolarctobacterium* were negatively associated with NMS scores, and genera *Amaricoccus*, *Bosea* *Janthinobacterium*, *Nesterenkonia* and *Sphingobacterium* were positively associated with HAMA scores. Additionally, genera *Aquabacterium*, *Bdellovibrio* and *Leucobacter* were positively associated with HAMD scores. No genus in the blood was associated with MMSE or MoCA scores (Table 4).

## Discussion

To date, identification of bacterial species is highly dependent on culture or molecular tests (Rudkjobing et al., 2016). Some of the limitations of culture methods are evident in the culturing of slow-growing or fastidious bacteria, which make identification a complicated and time/resource consuming process (Hasman et al., 2014). Nowadays, the progress of high-throughput sequencing and optimization of a specific pipeline of targeted metagenomics is a promising method to successfully quantify and characterize the taxonomic profile of the microbiome present in tissues, especially in blood. Many studies have reported that microbiota do exist in the blood of patients with some non-communicable diseases (Amar et al., 2011b, 2013; Potgieter et al., 2015; Lelouvier et al., 2016), even of healthy persons (Paisse et al., 2016). To our best knowledge, we think that our study is the first to investigate the association between the microbiota present in blood and neurological disorders. The gene copy numbers of no template controls used in our study is very low compared with that of blood. This supported that the microbiota in blood are not due to contamination from reagents. Additionally, the major phyla found in the healthy controls in our study are consistent with those of the healthy controls from previous studies worldwide (Lelouvier et al., 2016; Paisse et al., 2016). Considering unavoidable contamination, we paid more attention to the difference in the microbiota present in blood, which contributes to the identification of significant changes associated with the disease.

To our best knowledge, no exhaustive analysis of the microbiome in blood of patients with PD has been performed thus far. To address this gap, we performed 16S rRNA gene sequencing of microbiota DNA from the blood of PD patients and their healthy spouses. Different with the gut microbiota dysbiosis detected in PD patients, the structure and richness of the microbiota present in the blood were similar between PD patients and healthy controls. The increased level of *Cloacibacterium* in PD patients was also observed in the gastrointestinal tracts of patients with adenomas (Sanapareddy et al., 2012). The increased level of the genus *Microbacterium* in blood was associated with inflammation (Lau et al., 2002). Particularly, we designed genus-specific primers and performed real-time PCR to confirm the changes of these genus *Limnobacter*, *Myroides*, *Isoptericola*, *Microbacterium*, *Cloacibacterium* and *Enhydrobacter* in both PD patients and healthy controls. Furthermore, we tested these genera in the blood of another group of PD patients and healthy controls to validate the results. However, what is the biological significance of the association of certain genera with a specific disease is quite uncertain. Even different strains from the same species have a different impact on immune regulation (Sela et al., 2018), e.g., *Microbacterium* C448 showed bioremediation potential to regulate sulfonamide antibiotic biodegradation (Malcolm, 2017), while *Microbacterium* *nematophilum* could cause infection with an innate immune response (McMullan et al., 2012). Moreover, 15 genera in the microbiota of blood were identified in the RF model to predict the status of PD. The fecal microbiota community of these same 90 individuals were also detected by our team (Qian et al., 2018). The RF model also showed that the disease-specific alterations in the taxa in the blood had higher specificity than those of fecal microbiota (Scheperjans et al., 2015; Bedarf et al., 2017; Hopfner et al., 2017). Combining the taxa from the feces and blood, a higher AUC was identified to be predictive of PD, and this allowed us to develop a better integrated model than the use of fecal microbiota alone or models from other studies (Scheperjans et al., 2015; Bedarf et al., 2017; Hopfner et al., 2017). Thus, this may constitute a relevant and easy-to-use sampling approach for the diagnosis and characterization of PD. Furthermore, the microbiota related to novobiocin biosynthesis function was increased in the blood of PD patients. Novobiocin was reported to bind to the newly discovered Hsp90 C-terminal ATP binding site and induce degradation of Hsp90 client proteins, which was a major molecular chaperone responsible for the aggregation of alpha-synuclein (Donnelly and Blagg, 2008). In the future, shotgun metagenomic sequencing is necessary for the detailed analysis of the microbial taxon and function in the blood of PD patients.

To date, only one study including European patients with liver fibrosis has directly analyzed fecal and blood microbiota using 16S rDNA sequencing (Lelouvier et al., 2016). In our results, we found that the major components of the microbiota present in the blood differed largely from the microbiota detected in the feces, which was consistent with the results obtained by Lelouvier et al. (2016). Additionally, none of the significant taxa in blood associated with PD were shared with fecal microbiota. Taken together, our results do not support the hypothesis that the microbiota present in the blood originates from the gut microbiota as a result of bacterial translocation, which has been suggested for other noninfectious diseases, including cirrhosis (Bellot et al., 2010), diabetes (Amar et al., 2011a) and schizophrenia (Severance et al., 2013). Our results are consistent with a previous study on liver fibrosis (Lelouvier et al., 2016), which also showed that both the major component phyla and the disease-specific taxa differed between feces and blood. As the pathogenic blood microbiota has limited connections with the gut, the question remains as to where does it come from? Koren et al. (2011) suggested that oral bacteria might translocate into the blood in patients with atherosclerosis. Recently, the alteration of oral and nasal microbiota in PD patients were demonstrated (Heintz-Buschart et al., 2018; Pereira et al., 2017), but the microbiota in the blood were not investigated. Recently, a study analyzed the circulating microbiome in blood of different circulatory compartments in patients with liver cirrhosis and found that different genera existed in the different locations of the circulatory system (Schierwagen et al., 2018). This reminds us that investigations of the microbiome in not only the different circulatory compartments but also in the oral, blood, and gut associated with PD from the same subjects are needed in the future to explain the origin and role of microbiome in the blood. Antibiotics have been a topic of great interest in pre-clinical PD research for several years; however, studies of antibiotic function (e.g., ceftriaxone) have primarily focused on their neuroprotective properties, such as preventing toxic a-syn oligomer formation and ameliorating mitochondrial dysfunction (Reglodi et al., 2017) but have neglected their antimicrobial activity. Our results may suggest an important role of antimicrobial functions of the antibiotics used in PD.

In our study, we found that numerous bacteria were correlated with PD clinical characteristics, including disease duration, severity, medication, and non-motor symptoms. No taxa in the fecal microbiota were associated with H&Y stages and UPDRS total scores (Keshavarzian et al., 2015). Genus *Cloacibacterium* and* Isoptericola*, which were identified as significantly enriched in PD patients, also positively correlated with disease duration. Several genera in the blood were associated with UPDRS total scores, which indicated that these taxa in the blood may be sensitive in monitoring disease severity. Moreover, Hill-Burns et al. (2017) showed the independent effects of PD medications on the gut microbiome. In the blood, we found that some taxa were associated with LED, demonstrating that PD medications might affect the blood microbiota or the microbiota might influence drug metabolism (Hill-Burns et al., 2017). In our previous study, some taxa in the feces were identified as associated with motor complications. In this study, genus *Dokdonella* in the blood was identified to be associated with motor complications, which might hint that the role of certain bacteria in their occurrence at different tissues. Moreover, we identified a correlation between microbiota in the blood with NMSs. Our results indicated firstly that several taxa in the blood were associated with depression or anxiety, even though this relationship has not been discovered before.

### Limitations

Our study has some limitations. Considering the limitation of the sample size, larger research studies in other populations are needed to confirm our results on a large scale. Furthermore, our study is a cross-sectional study. Longitudinal studies that focus on the microbiota in the blood at different periods in PD patients are needed. Particularly, as the blood is no longer to be recognized as “sterile,” the next target of microbiota study in PD is the brain. It is necessary to investigate the microbiota in oral cavity, blood, gut, and even cerebrospinal fluid using 16S rRNA gene metagenome analysis or even better methods such as shotgun sequencing to provide more knowledge of the role of the microbiota in the pathogenesis of PD.

## Conclusion

We discovered for the first time that not only gut microbiota dysbiosis but also the alterations of the microbial 16S rRNA gene in the blood were associated with PD. Furthermore, the bacteria present in the blood was more closely related to PD clinical characteristics. Elucidating these differences in the blood microbiomes will provide a foundation to improve our understanding of the role of the microbiota in the pathogenesis of PD, and the potential roles of microbiota from different tissues need further consideration.

## Data Availability

The high-throughput sequence data have been deposited in the National Center for Biotechnology Information (NCBI) BioProject database with project number PRJNA391524. All other data are available upon request from the authors.

## Author Contributions

YQ and XY: clinical analyses and manuscript writing. SX: sample collection and DNA extraction. CW: statistical analyses and sequencing. NQ: sequencing analyses and management. S-DC: project supervision and manuscript revision. QX: study design, project management, major financial support and manuscript revision.

## Conflict of Interest Statement

The authors declare that the research was conducted in the absence of any commercial or financial relationships that could be construed as a potential conflict of interest.

## Footnotes

^1^http://huttenhower.sph.harvard.edu/lefse/

^2^http://www.genome.jp/kegg/pathway.html
