# Corrigendum: Detection of Microbial 16S rRNA Gene in the Blood of Patients With Parkinson's Disease

**DOI:** 10.3389/fnagi.2019.00004

**Published:** 2019-01-30

**Authors:** Yiwei Qian, Xiaodong Yang, Shaoqing Xu, Chunyan Wu, Nan Qin, Sheng-Di Chen, Qin Xiao

**Affiliations:** ^1^Department of Neurology & Collaborative Innovation Center for Brain Science, Ruijin Hospital, Shanghai Jiao Tong University School of Medicine, Shanghai, China; ^2^Department of Bioinformatics, Realbio Genomics Institute, Shanghai, China

**Keywords:** neurodegeneration disease, blood, microbiota, 16S rRNA gene, inflammation

Due to a flaw in analysis, there was a technical error used in the following classical formula to calculate copy numbers.

“Number of copies = (amount of DNA (ng) ^*^ 6.022 x 10^23^) / (length ^*^ 1 x 10^9^^*^ 650) (650 = Molecular weight/bp, 6.022 ^*^10^23^ = Avogadro' s number).”

The length of 16S rRNA gene of *Escherichia coli* BL21 strain, which is “1,542 bp,” instead of the length of its genome, which is “4,558,947 bp,” was used to match the amount of DNA of this strain. This led to the errors regarding the 16S gene (copies/ng) of the PD and healthy group in the Table 2, Supplementary Figure S1 and a statement in the Results in the original article.

The corrected Table 2 and Supplementary Figure S1 appear below.

**Table 2:** Characteristics of the study subjects.

**Characteristics**	**PD group (*n* = 45)**	**Healthy group (*n* = 45)**	***P-*value**
Age (years)^a^	68.1 (8.0)	67.9 (8.0)	0.875
Female (n, %)	23 (51.1%)	22 (48.9%)	0.903
BMI (kg/m^2^)^a^	22.8 (2.3)	23.4 (2.1)	0.164
Constipation (n, %)	24 (53.3%)	3 (6.7%)	< 0.001
16s gene (copies/ng)^b^	1.22E+04 (1.21E+05)	7.78E+03 (6.86E+04)	0.316

*Data are shown as mean (SD)^a^ or median (IQR)^b^. Comparisons between the two groups were performed with Student's t-test (age and BMI) and Pearson's Chi-square test (female and constipation), respectively. The 16S gene copies were normalized to 1 ng of each DNA sample, and the comparison between the two groups was performed with Wilcoxon rank-sum test analysis. PD, Parkinson's disease; BMI, Body Mass Index*.

**Figure S1:**
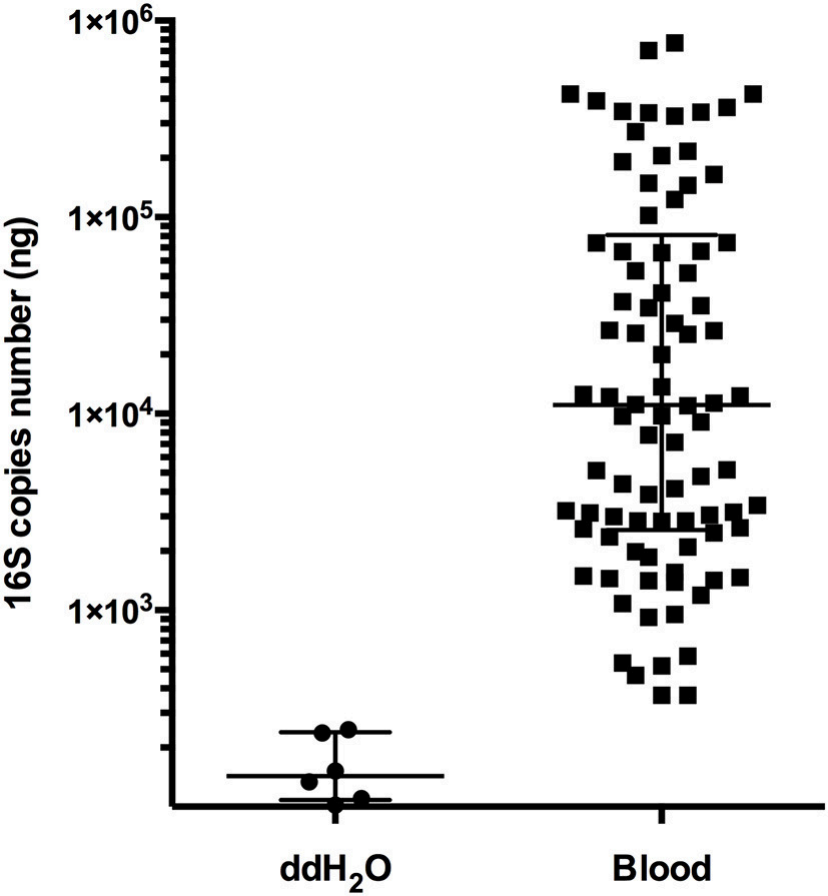
Comparison the 16S gene copies from the no template control and blood samples from 90 individuals by real-time PCR analysis.

A correction has also been made to the **Results**, **Characteristics of the Studied Groups**:

“There was no significant difference in the 16S rRNA gene copies between PD and healthy groups by real-time PCR (1.22E+04 ± 1.21E+05 copies/ng of PD vs. 7.78E+03 ± 6.86E+04 copies/ng of controls, *P* = 0.316, Wilcoxon rank-sum test analysis).”

The authors apologize for this error and state that this does not change the scientific conclusions of the article in any way. The original article has been updated.

## Conflict of Interest Statement

The authors declare that the research was conducted in the absence of any commercial or financial relationships that could be construed as a potential conflict of interest.

