# Supplementary material for: Detection of Microbial 16S rRNA Gene in the Blood of Patients With Parkinson’s Disease
Source: Front Aging Neurosci. 2018 May 24;10:156. doi: 10.3389/fnagi.2018.00156 (PMC5976788; doi:10.3389/fnagi.2018.00156)
Supplement: Supplementary file 1 [file Table_1.DOCX]

**Table S1. Differences of the microbiota at all levels in blood between PD and healthy groups**

| **Taxonomic Level** | **mean(D)** | **mean(H)** | ***P* ^a^** | **FDR, *P* ^b^** |
| --- | --- | --- | --- | --- |
| Order | | | | |
| Proteobacteria; Aeromonadales | 0.0328% | 0.0032% | 0.0039 | 0.5036 |
| Proteobacteria; Bdellovibrionales | 0.0513% | 0.0897% | 0.0194 | 0.8419 |
| Family | | | | |
| Actinobacteria; Actinomycetaceae | 0.0208% | 0.0050% | 0.0206 | 0.8419 |
| Proteobacteria; Aeromonadaceae | 0.0328% | 0.0032% | 0.0039 | 0.5036 |
| Firmicutes; Bacillales Incertae Sedis XI | 0.0027% | 0.0031% | 0.0282 | 0.9207 |
| Proteobacteria; Bdellovibrionaceae | 0.0389% | 0.0819% | 0.0336 | 0.9207 |
| Firmicutes; Clostridiaceae 1 | 0.0230% | 0.0457% | 0.0436 | 0.9207 |
| Firmicutes; Enterococcaceae | 0.0146% | 0.0167% | 0.0380 | 0.9207 |
| Actinobacteria; Promicromonosporaceae | 0.0490% | 0.0099% | 0.0355 | 0.9207 |
| Actinobacteria; Propionibacteriaceae | 0.0555% | 0.0117% | 0.0005 | 0.3027 |
| Firmicutes; Staphylococcaceae | 0.0336% | 0.0175% | 0.0115 | 0.7091 |
| Actinobacteria; Streptomycetaceae | 0.0172% | 0.0114% | 0.0395 | 0.9207 |
| Actinobacteria; Thermomonosporaceae | 0.0011% | 0.0042% | 0.0479 | 0.9207 |
| Proteobacteria; Xanthobacteraceae | 0.0091% | 0.0034% | 0.0404 | 0.9207 |
| Genus | | | | |
| Actinobacteria; Actinomyces (Actinomycetaceae) | 0.0208% | 0.0040% | 0.0054 | 0.5036 |
| Bacteroidetes; Cloacibacterium (Flavobacteriaceae) | 0.0324% | 0.0085% | 0.0062 | 0.5036 |
| Proteobacteria; Dokdonella (Xanthomonadaceae) | 0.0015% | 0.0054% | 0.0440 | 0.9207 |
| Proteobacteria; Enhydrobacter (Moraxellaceae) | 0.0326% | 0.0094% | 0.0264 | 0.9207 |
| Firmicutes; Enterococcus (Enterococcaceae) | 0.0134% | 0.0167% | 0.0380 | 0.9207 |
| Firmicutes; Gemella (Bacillales_Incertae Sedis XI) | 0.0027% | 0.0031% | 0.0282 | 0.9207 |
| Actinobacteria; Isoptericola (Promicromonosporaceae) | 0.0440% | 0.0042% | 0.0124 | 0.7091 |
| Proteobacteria; Jahnella (Polyangiaceae) | 0.0028% | 0.0008% | 0.0300 | 0.9207 |
| Proteobacteria; Limnobacter (Burkholderiaceae) | 0.2069% | 0.3408% | 0.0484 | 0.9207 |
| Actinobacteria; Microbacterium (Microbacteriaceae) | 0.0730% | 0.0408% | 0.0194 | 0.8419 |
| Bacteroidetes; Myroides (Flavobacteriaceae) | 0.0459% | 0.0142% | 0.0045 | 0.5036 |
| Actinobacteria; Propionicimonas (Propionibacteriaceae) | 0.0032% | 0.0003% | 0.0121 | 0.7091 |
| Firmicutes; Staphylococcus (Staphylococcaceae) | 0.0302% | 0.0115% | 0.0022 | 0.5036 |
| Actinobacteria; Streptomyces (Streptomycetaceae) | 0.0157% | 0.0097% | 0.0317 | 0.9207 |
| Firmicutes; Trichococcus (Carnobacteriaceae) | 0.0137% | 0.0044% | 0.0165 | 0.8419 |

() family name of each genus

^a^ Wilcoxon rank-sum test analysis

^b^ Benjamini-Hochberg false discovery rate (FDR-P)-corrected P value

D, PD group; H, healthy group.
