# Supplementary material for: Detection of Microbial 16S rRNA Gene in the Blood of Patients With Parkinson’s Disease
Source: Front Aging Neurosci. 2018 May 24;10:156. doi: 10.3389/fnagi.2018.00156 (PMC5976788; doi:10.3389/fnagi.2018.00156)
Supplement: Supplementary file 2 [file Table_2.DOCX]

**Table S2. Differences of the microbiota at all levels in blood between patients with and without motor complications**

| **blood** | **Mean**  **(MC, n=16)** | **Mean**  **(NMC, n=29)** | ***P* ^a^** | **FDR, *P* ^b^** |
| --- | --- | --- | --- | --- |
| **Class** |  |  |  |  |
| Anaerolineae | 0.0023% | 0.0006% | 0.039 | 0.991 |
| **Order** |  |  |  |  |
| Aeromonadales | 0.0261% | 0.0365% | 0.035 | 0.991 |
| Anaerolineales | 0.0023% | 0.0006% | 0.039 | 0.991 |
| Chloroflexales | 0.0042% | 0.0025% | 0.032 | 0.991 |
| **Family** |  |  |  |  |
| Actinomycetaceae | 0.0325% | 0.0144% | 0.022 | 0.991 |
| Aeromonadaceae | 0.0261% | 0.0365% | 0.035 | 0.991 |
| Anaerolineaceae | 0.0023% | 0.0006% | 0.039 | 0.991 |
| Campylobacteraceae | 0.0162% | 0.0004% | 0.010 | 0.991 |
| **Genus** |  |  |  |  |
| Actinomyces | 0.0325% | 0.0144% | 0.022 | 0.991 |
| Chlorophyta | 0 | 0.0025% | 0.037 | 0.991 |
| Dokdonella | 0.0030% | 0.0006% | 0.014 | 0.991 |
| Megasphaera | 0.0034% | 0.0367% | 0.034 | 0.991 |

MC, motor complication; NMC, no motor complication

P, phylum; c, class; o, order; f, family; g, genus.

^a^ Wilcoxon rank-sum test analysis

^b^ Benjamini-Hochberg false discovery rate (FDR-P)-corrected P value
