# Supplementary material for: Detection of Microbial 16S rRNA Gene in the Blood of Patients With Parkinson’s Disease
Source: Front Aging Neurosci. 2018 May 24;10:156. doi: 10.3389/fnagi.2018.00156 (PMC5976788; doi:10.3389/fnagi.2018.00156)
Supplement: Supplementary file 3 [file Table_3.DOCX]

**Table S3. Reference strains of each genus used for real-time PCR in this study**

| genus | species |
| --- | --- |
| *Cloacibacterium* | *Cloacibacterium rupense* NBRC 104931;  *Cloacibacterium normanense* CCUG 46293;  *Cloacibacterium normanense* isolate Bug15;  *Cloacibacterium haliotis* WB5;  *Cloacibacterium* sp. B6;  *Cloacibacterium* sp. C10-92;  *Cloacibacterium* sp. GENT20;  *Cloacibacterium* sp. R091;  Uncultured *Cloacibacterium* sp. isolate OTU1294;  Uncultured *Cloacibacterium* sp. isolate 16T_24805;  Uncultured *Cloacibacterium* sp. isolate 2_37H2OII |
| *Enhydrobacter* | *Enhydrobacter aerosaccus* PAGU 1700;  *Enhydrobacter aerosaccus* DSR16;  *Enhydrobacter aerosaccus* DNF00742;  *Enhydrobacter aerosaccus* 30.3.1;  *Enhydrobacter* sp. H7;  *Enhydrobacter* sp. ITCr12;  *Enhydrobacter* sp. N1-7a;  *Enhydrobacter* sp. KB3-12;  *Enhydrobacter* sp. MadaFrogSkinBac. DB-.1233;  *Enhydrobacter* sp. Td-10;  *Enhydrobacter* sp. C8-1;  *Enhydrobacter* sp. M_Sw_oHS_07/11_2_2(1);  *Enhydrobacter* sp. DV9-3;  Uncultured *Enhydrobacter* sp. clone MZ4P9S26-c47;  Uncultured *Enhydrobacter* sp. clone JU-GC3;  Uncultured *Enhydrobacter* sp. isolate 45N_3167;  Uncultured *Enhydrobacter* sp. clone HNA1_290108_7 |
| *Isoptericola* | *Isoptericola variabilis* CE85;  *Isoptericola variabilis* MX5;  *Isoptericola jiangsuensis* CLG;  *Isoptericola nanjingensis* H17;  *Isoptericola rhizophila* BKS 3-46;  *Isoptericola cucumis* AP-38;  *Isoptericola dokdonensis* DS-3;  *Isoptericola hypogeus* NBRC 104396;  *Isoptericola halotolerans* NBRC 104116;  *Isoptericola variabilis* NBRC 104115;  *Isoptericola chiayiensis* isolate SR4-57;  *Isoptericola salitolerans* TSTB0-51;  *Isoptericola* sp. FS53;  *Isoptericola* sp. MS26;  *Isoptericola* sp. CC 0320;  *Isoptericola* sp. TUT1258;  *Isoptericola* sp. KLBMP S0046;  *Isoptericola* sp. JC408;  *Isoptericola* sp. RM478;  *Isoptericola* sp. JC138, isolate A2;  *Isoptericola* sp. OS-B31 |
| *Limnobacter* | *Limnobacter litoralis* KP1-19;  *Limnobacter thiooxidans* isolate 0312MAR12L4  *Limnobacter thioxidans* TSWCSN35;  *Limnobacter humi* UCM-39;  *Limnobacter* sp. AK108, isolate A2a-2;  *Limnobacter* sp. KP2-69;  *Limnobacter* sp. KP1-80;  *Limnobacter* sp. AK51  *Limnobacter* sp. F3;  Uncultured *Limnobacter* sp. clone 133X76C_27  Uncultured *Limnobacter* sp. clone Flu2_11;  Uncultured *Limnobacter* sp. clone Puga_TWC_XXX383 |
| *Myroides* | *Myroides injenensis* M09-1053;  *Myroides xuanwuensis* TH-19;  *Myroides xuanwuensis* VBN31;  *Myroides marinus* JS-08;  *Myroides odoratimimus* FFA2;  *Myroides odoratimimus* CCUG 39352T;  *Myroides pelagicus*;  *Myroides pelagicus* NBRC 102469;  *Myroides guanonis* IM13T;  *Myroides gitamensis* BSH-3T;  *Myroides odoratus* HAMBI 1923;  *Myroides phaeus* MY15;  *Myroides* sp. ACR3;  *Myroides indicus* UKS3 |
| *Microbacterium* | *Microbacterium sediminicola*;  *Microbacterium takaoensis*;  *Microbacterium schleiferi*;  *Microbacterium keratanolyticum*;  *Microbacterium flavescens*;  *Microbacterium arabinogalactanolyticum*;  *Microbacterium terricola*;  *Microbacterium fluvii*;  *Microbacterium ketosireducens*;  *Microbacterium terregens*;  *Microbacterium saperdae*;  *Microbacterium luteolum*;  *Microbacterium aurantiacum*;  *Microbacterium trichotecenolyticum*;  *Microbacterium aoyamense*;  *Microbacterium pumilum*;  *Microbacterium deminutum*;  *Microbacterium hominis*;  *Microbacterium chocolatum*;  *Microbacterium arborescens*;  *Microbacterium awajiense*;  *Microbacterium pygmaeum* |
