# Supplementary material for: Detection of Microbial 16S rRNA Gene in the Blood of Patients With Parkinson’s Disease
Source: Front Aging Neurosci. 2018 May 24;10:156. doi: 10.3389/fnagi.2018.00156 (PMC5976788; doi:10.3389/fnagi.2018.00156)
Supplement: Supplementary file 4 [file Data_Sheet_1.PDF]

Supplementary Material

Detection the microbial 16S rRNA gene in the blood of patients with Parkinson's disease

Yiwei Qian, Xiaodong Yang, Shaoqing Xu, Chunyan Wu, Nan Qin*, Sheng-Di Chen*, Qin Xiao*

*** Correspondence:**
Nan Qin (qinnan@gmail.com), Sheng-Di Chen (ruijincsd@126.com) and Qin Xiao (xq10537@rjh.com.cn)

Supplementary Figures

Figure S1. Comparison the 16S gene copies from the no template control and blood samples by real-time PCR analysis
Figure S2. The α-diversity and β-diversity indices of the microbiomes in blood between the PD and healthy groups

Supplementary Tables

Table S1. Differences of the microbiota at all levels in blood between PD and healthy groups
Table S2. Differences of the microbiota at all levels in blood between patients with and without motor complications
Table S3. Reference strains of each genus used for real-time PCR in this study

Supplementary Figures

Figure S1. Comparison the 16S gene copies from the no template control and blood samples from 90 individuals by real-time PCR analysis.

Scatter plots showed the 16S gene copies in the negative controls and blood from 90 individuals at the same time by real-time PCR (negative controls were molecular grade water added in an empty tube, extracted and analyzed at the same time as the samples). Each bar represents the median and interquartile range, respectively.

Figure S2. The α-diversity and β-diversity indices of the microbiomes in blood between the PD and healthy groups.

(A) Box plots depict differences in the blood microbiome diversity indices between the PD and healthy groups according to the Chao 1 index, observed species index, PD whole tree index, Shannon index and Simpson index based on the OTU counts. Each box plot represents the median, interquartile range, minimum, and maximum values.

(B) Unweighted and weighted ANOSIMs and PCOA based on the distance matrix of UniFrac dissimilarity of the blood microbial communities in the PD and healthy groups. Respective ANOSIM R values show the community variation between the compared groups, and significant P values are indicated. The axes represent the two dimensions explaining the greatest proportion of variance in the communities. Each symbol represents a sample, and each line connects a pair of samples.

D, PD group (blue); H, healthy group (red).

OTU, operational taxonomic unit; ANOSIM, analyses of similarities; PCOA, principal coordinates analysis.
