# Supplementary figures and images for: Detection of Microbial 16S rRNA Gene in the Blood of Patients With Parkinson’s Disease
Source: Front Aging Neurosci. 2018 May 24;10:156. doi: 10.3389/fnagi.2018.00156 (PMC5976788; doi:10.3389/fnagi.2018.00156)

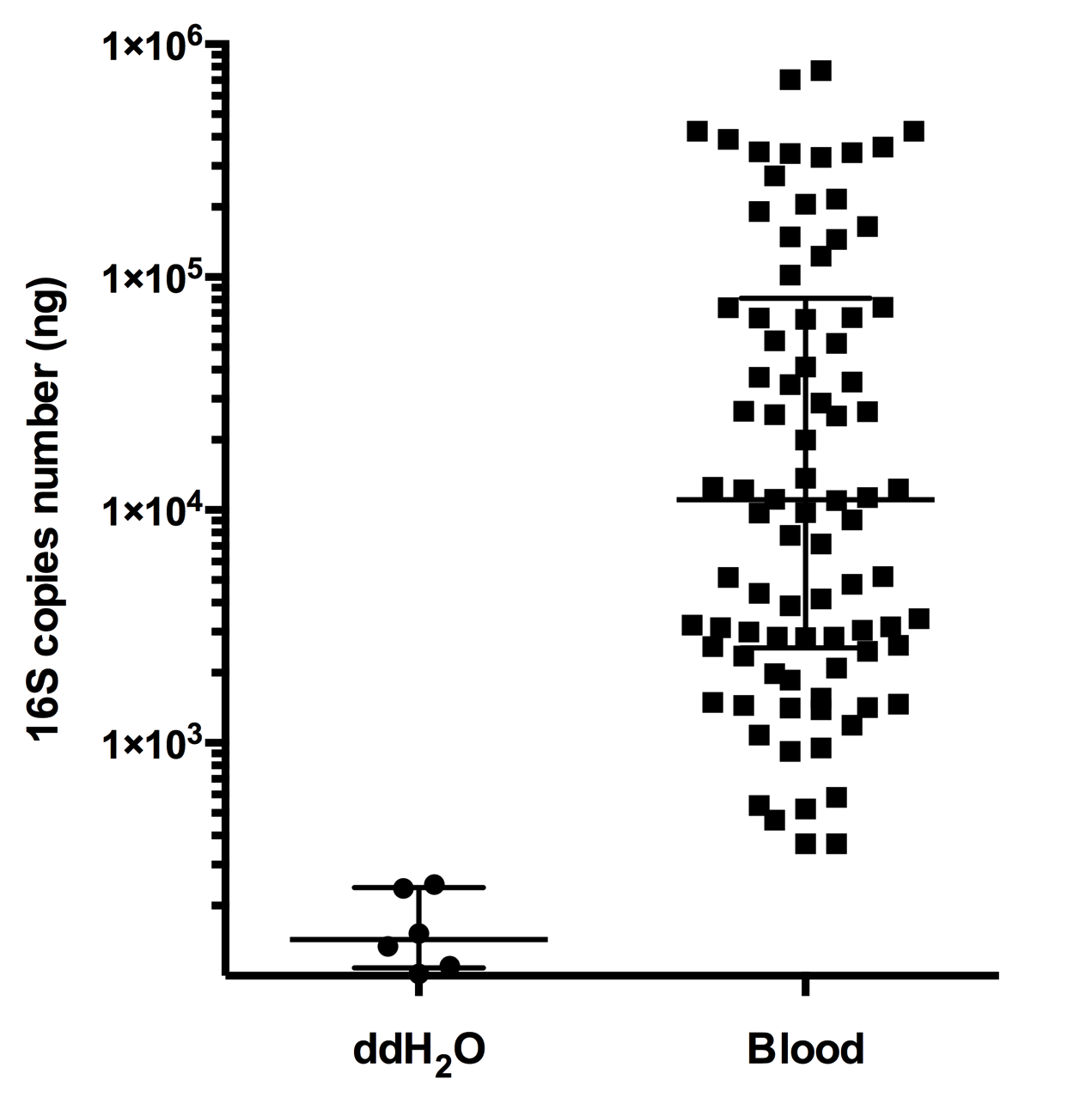

Supplement: Supplementary file 5 [file Image_1.TIFF]

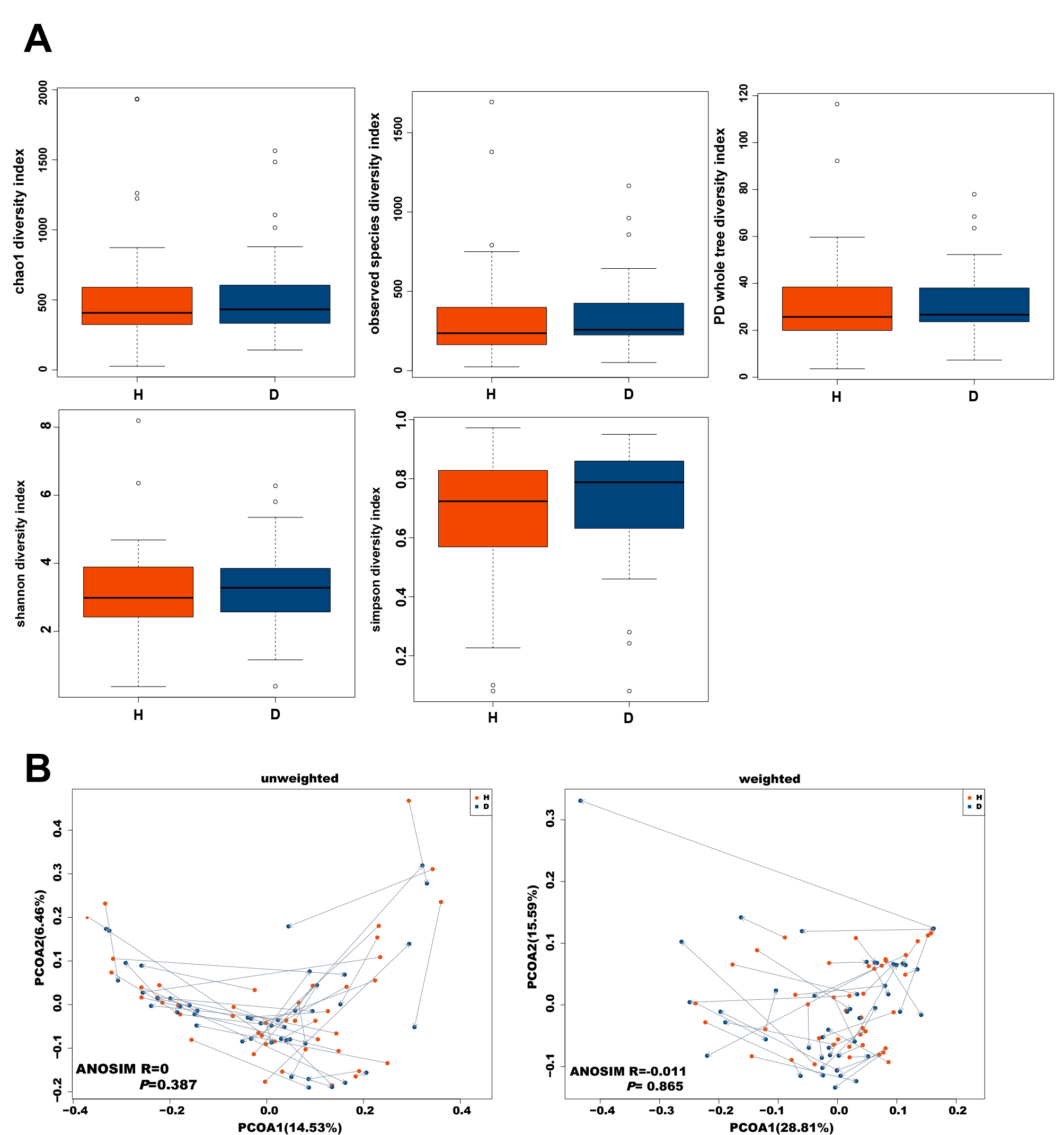

Supplement: Supplementary file 6 [file Image_2.TIF]
